# Enhanced Cytotoxic Effect of TAT–PLGA-Embedded DOXO Carried by Biomimetic Magnetic Nanoparticles upon Combination with Magnetic Hyperthermia and Photothermia

**DOI:** 10.3390/pharmaceutics13081168

**Published:** 2021-07-28

**Authors:** Ylenia Jabalera, Alberto Sola-Leyva, Salvatore Calogero Gaglio, María P. Carrasco-Jiménez, Guillermo R. Iglesias, Massimiliano Perduca, Concepcion Jimenez-Lopez

**Affiliations:** 1 Department of Microbiology, Faculty of Sciences, University of Granada, 18071 Granada, Spain; yjabalera@ugr.es; 2 Department of Biochemistry and Molecular Biology I, University of Granada, 18071 Granada, Spain; albertosola@ugr.es; 3 Instituto de Investigación Biosanitaria ibs.Granada, 18014 Granada, Spain; 4 Department of Biotechnology, University of Verona, Strada Le Grazie 15, 37134 Verona, Italy; salvatorecalogero.gaglio@univr.it; 5 Department of Applied Physic, Faculty of Sciences, University of Granada, 18071 Granada, Spain

**Keywords:** photothermia, hyperthermia, BMNPs, PLGA, magnetic nanoparticles, TAT peptide, doxorubicin

## Abstract

The synergy between directed chemotherapy and thermal therapy (both magnetic hyperthermia and photothermia) mediated by a nanoassembly composed of functionalized biomimetic magnetic nanoparticles (BMNPs) with the chemotherapeutic drug doxorubicin (DOXO) covered by the polymer poly(lactic-*co*-glycolic acid) (PLGA), decorated with TAT peptide (here referred to as TAT–PLGA(DOXO-BMNPs)) is explored in the present study. The rationale behind this nanoassembly lies in an optimization of the nanoformulation DOXO-BMNPs, already demonstrated to be more efficient against tumor cells, both in vitro and in vivo, than systemic traditional therapies. By embedding DOXO-BMNPs into PLGA, which is further functionalized with the cell-penetrating TAT peptide, the resulting nanoassembly is able to mediate drug transport (using DOXO as a drug model) and behaves as a hyperthermic agent (induced by an alternating magnetic field (AMF) or by laser irradiation with a laser power density of 2 W/cm^2^). Our results obtained using the HepG2 cell line show that there is a synergy between chemotherapy and thermal therapy that results in a stronger cytotoxic effect when compared to that caused by the soluble DOXO. This is probably due to the enhanced DOXO release occurring upon the application of the thermal therapy, as well as the induced local temperature rise mediated by BMNPs in the nanoassembly following exposition to AMF or to near-infrared (NIR) laser irradiation. These results represent a proof of concept demonstrating that TAT–PLGA(DOXO-BMNPs) can be used to efficiently combine therapies against tumor cells, which is a step forward in the transition from systemic to local treatments.

## 1. Introduction

MamC-mediated biomimetic magnetic nanoparticles (BMNPs) have been recently proposed as promising drug nanocarriers and hyperthermic agents due to their novel properties inferred by the mediation of the *Magnetococcus marinus* MC-1 magnetosome protein MamC on the formation of the nanoparticles [1,2,3]. Like many other magnetic nanoparticles, BMNPs are biocompatible; their large surface area allows the carrying of relatively large amounts of the relevant molecule, they can be externally directed to the target site following the application of a magnetic field, and they have been shown to be hyperthermic agents [4,5,6]. Moreover, BMNPs overcome two main drawbacks of commercialized magnetic nanoparticles (MNPs): one is linked to the relatively small size of MNPs (~10–20 nm), which results in a small magnetic moment per particle, and the other is related to the surface charge of MNPs (fairly low (or neutral) at physiological pH [3,6,7,8]), which requires a further coating to allow functionalization [6] that may shelter the already nonoptimal magnetic properties of those nanoparticles [8]. The presence of MamC in BMNPs confers surface functional groups to the nanoparticle that allow functionalization without the need of a further coating [3]. Moreover, the control that MamC exerts over the nucleation and growth of the crystals results in the formation of magnetite crystals of about 40 nm in size. This size increase with respect to the synthetic ones is enough to increase the magnetic moment per particle while maintaining their superparamagnetism (i.e., they behave as nonmagnetic in the absence of an external magnetic field, but respond with a maximized magnetic susceptibility once an external magnetic field is applied) [9]. Furthermore, BMNPs were demonstrated to increase the temperature following the application of an alternating magnetic field (AMF), which can be used not only to locally increase the temperature to the so-called therapeutic hyperthermic temperature (43–46 °C), but also to further increase drug release [10,11,12]. Another mechanism also capable of inducing temperature elevation is called photothermal therapy, in which nanoparticles, when irradiated with a near-infrared (NIR) laser, absorb electromagnetic energy and convert it into heat. This makes it possible to produce hyperthermia with low laser powers and low concentrations of nanoparticles [13,14,15,16]. The individual application of magnetic and photothermal hyperthermia is currently being tested and studied. Each of the individual treatments produces both a drug-releasing effect and a local increase in temperature when the concentration is optimal, and many authors are combining both techniques, demonstrating a synergistic effect of a combined therapy [17,18,19].

However, there are still some drawbacks that require further optimization of the BMNP-mediated therapy. Specifically, this optimization needs to improve cell uptake and colloidal stability, which, in turn is expected to increase the efficiency of the treatment. In particular, the nanoassembly composed of doxorubicin (DOXO) and BMNPs, named DOXO-BMNPs, has already proven its effectiveness against tumor cells both in vitro [3] and in vivo [20], by mediating directed chemotherapy and acting as a magnetic hyperthermic agent. However, since DNA is the target of DOXO, the effectiveness of the DOXO-BMNP nanoassembly depends upon the nanoassembly uptake by cells and/or DOXO diffusion to the cell nuclei.

A way to improve such an internalization is to embed the nanoassembly in liposomes or polymeric nanoparticles [21,22]. Among them, the use of poly(lactic-*co*-glycolic acid) (PLGA) is particularly interesting due to its biocompatibility and biodegradability. PLGA is hydrolyzed by cells to produce the original monomers, lactic acid and glycolic acid, two subproducts with very low systemic toxicity, which finally undergo biodegradation through the Krebs cycle. In fact, PLGA is approved for human therapies both by the Food and Drug Administration (FDA) and the European Medicines Agency [23,24,25]. Another advantage in using PLGA nanoparticle technology is the presence of carboxyl groups on its surface, useful for functionalization with signal molecules to archive targeting and specific delivery of the cargo. A recent example is represented by the synthesis of hyaluronic acid-derivatized PLGA nanoparticles to actively target the CD44 receptors present on the surface of breast cancer cells and selectively delivering the antitumoral molecule tamoxifen, well known to be active against estrogen receptor-positive breast cancer cells, thus reducing the correlated side-effects [26]. Among the different peptides implemented as targeting molecules in nanoparticle development, an important role is represented by the HIV transcriptional activator protein TAT peptide (TAT), which has been widely used as an uptake enhancer for cancer cells [27,28].

PLGA has also been used in the recent past to encapsulate MNPs [29], as well BMNPs [22]. In this second study, we demonstrated that PLGA and/or TAT–PLGA covering of BMNPs does improve the cell uptake of the nanoparticles, does not shelter the magnetic properties of the BMNPs, and maintains their ability to behave as hyperthermic agents. However, in that study, the potential of the nanoassembly to induce a synergy between directed chemotherapy and the magnetic hyperthermic agent was not studied, since BMNPs were not functionalized. Moreover, the potential of these TAT–PLGA-embedded DOXO-BMNPs to mediate synergy between chemotherapy and photothermia, as an alternative therapy to locally increase the temperature and/or facilitate drug release at the target, has not yet been explored. Therefore, in the present study, we aimed to optimize the effectiveness of the DOXO-BMNP nanoassembly, using DOXO as a model drug molecule, by exploring different approaches. On one hand, this optimization was attempted by embedding DOXO-BMNPs into PLGA nanoparticles functionalized with TAT peptide with the goal of increasing cell uptake and, on the other hand, by combining this optimized directed chemotherapy with magnetic hyperthermia and photothermia.

## 2. Materials and Methods

### 2.1. BMNP Production

MamC was expressed and purified as a recombinant protein following the protocol previously described in [1]. Briefly, transformed *Escherichia coli* TOP10 cells (Life Technologies: Invitrogen, Grand Island, NY, USA) were grown at 37 °C in Luria–Bertani (LB) broth supplemented with ampicillin, and the expression of the MamC protein was induced with isopropyl-β-d-thiogalactopyranoside (IPTG, Fisher BioReagents, Pittsburgh, PA, USA). Once expressed, the purification of the protein was carried out under denaturing conditions by fast protein liquid chromatography (FPLC, GE Healthcare) using immobilized metal affinity chromatography (IMAC, GE Healthcare, Chicago, IL, USA). Lastly, fractions containing MamC were refolded at 4 °C through dialysis.

The synthesis of BMNPs was carried out at 1 atm total pressure and 25 °C from oxygen-free solutions, at a pH value of 9, following the protocol described in [1,30]. All experiments were done under anaerobic conditions inside an anaerobic Coy chamber (96% N_2_/4% H_2_, Coy Laboratory Products, Grass Lake, MI, USA). Samples were incubated for 30 days, and then the solids, following magnetic concentration and discarding of the supernatant, were washed with deoxygenated Milli-Q water. Lastly, the solid precipitated was stored in HEPES buffer (HEPES 10 mM, NaCl 150 mM, pH 7.4) and sterilized. These BMNPs were previously characterized in [3,16], and they were shown to consist of pure stoichiometric magnetite with an average size of 39 ± 7 nm (sizes from 10 to 70 nm) and an isoelectric point (iep) of ~4.1. BMNPs exhibit a high absorbance in the near-infrared region (NIR), as determined by UV−Vis−NIR absorbance spectra, due to the electronic transitions between Fe^2+^ and Fe^3+^.

### 2.2. BMNP Functionalization

BMNPs were functionalized with the chemotherapeutic drug DOXO (Sigma-Aldrich, Madrid, Spain), following the protocol described in [3,12]. Briefly, 5 mg of BMNPs were mixed with 1 mg/mL DOXO suspended in HEPES buffer at pH 7.4 for 24 h. Then, the nanoassemblies were magnetically collected and washed three times with HEPES buffer. The amounts of DOXO from the supernatants and the washings were quantified by UV–Vis spectroscopy (λ = 490 nm). This measured unbound DOXO was subtracted from the amount initially incubated, so that the amount of bound DOXO and the functionalization efficiency could be indirectly determined. The functionalization efficiency was 60% ± 10% for DOXO, in agreement with previous results [3,11]. These nanoassemblies were further characterized, showing sizes (measured by dynamic light scattering) of 160 ± 30 nm, an iep of ~6, and a high absorbance in the NIR region, also in agreement with our previous findings [16].

### 2.3. PLGA Encapsulation of BMNPs and DOXO-BMNPs, and TAT Peptide Functionalization

The encapsulation protocol for the BMNPs and DOXO-BMNP nanoassemblies is based on the single emulsion-evaporation method [22,31]. To prevent the oxidation of the magnetic particles, all buffers and aqueous solutions were degassed under vacuum. Then, 10 mg of the PLGA copolymer (50:50) was dissolved in 1 mL of organic solution (85% acetone and 15% ethanol) together with 5 mg of BMNPs or DOXO-BMNP nanoassemblies. The mixture was dripped in 10 mL of sterile PVA (1%) in a sonication bath, keeping the sonication intensity constant. The suspension mixture was stirred at 20 °C overnight under nitrogen atmosphere to evaporate the organic phase. The preparation was recovered using a magnet, and the pellet was washed three times with sterile filtered phosphate-buffered saline (PBS) solution, pH 7.4. The final pellet was resuspended in 1 mL of sterile PBS buffer and stored at 4 °C. In the case of DOXO-BMNPs, the efficiency of DOXO encapsulation was indirectly measured from the supernatants as detailed above. The encapsulation efficiency was 50% ± 15%.

To functionalize the PLGA surface of the nanoassemblies with the TAT peptide, 5 mg of the previously prepared PLGA(DOXO-BMNPs) or PLGA(BMNPs) were resuspended in 1 mL of 50 mM 2-(*N*-morpholino)ethanesulfonic acid (MES; Merck KGaA, Darmstadt, Germany), pH 5.8, and subsequently activated with 0.1 M 1-ethyl-3–(3-dimethylaminopropyl)carbodiimide (EDC; Merck KGaA, Darmstadt, Germany) and 0.7 M *N*-hydroxysuccinimide (NHS; Merck KGaA, Darmstadt, Germany) for 1 h at 20 °C. Then, the nanoassemblies were recovered using a magnet and washed with PBS. Lastly, 250 μg of TAT peptide were added to the suspension and incubated overnight at 20 °C under shaking. The reaction was stopped by adding glycine (20 mg/mL) and leaving the reaction at room temperature for 1 h. The functionalized nanoassemblies were subsequently washed with PBS, and the final pellet was resuspended in 1 mL of sterile PBS buffer and stored at 4 °C. TAT–PLGA embedding of BMNPs was previously shown to shelter neither the magnetic susceptibility of BMNPs (~56 emu/g for both BMNPs and TAT–PLGA(BMNPs) nor the potential of BMNPs to behave as hyperthermic agents.

### 2.4. TAT–PLGA(DOXO-BMNPs) Nanoassembly Characterization

The TAT–PLGA(DOXO-BMNPs) nanoassembly was characterized by nanoparticle tracking analysis (NTA) using a Malver NanoSight NS300 instrument on diluted samples (1:1000) at 25 °C. For simplicity, the results obtained for this nanoassembly were compared to those obtained from PLGA(BMNPs), as previous results demonstrated that the TAT peptide did not significantly increase the size of the nanoassembly compared to that of PLGA(BMNP) by ~7% [22]. For each sample, three sequences of 30 s with 25 FPS were recorded. The data analysis was carried out using NTA 3.4 Build 3.4.003 software. For the atomic force microscopy (AFM) analysis, 20 μL drops of each suspension sample (previously sonicated for 5 min) were deposited on 20 mm diameter mica discs, and the excess solvent was allowed to evaporate at room temperature. An NT-MDT Solver Pro microscope (Moscow, Russia), with a single-crystal silicon–antimony-doped probe and a gold-coated tip (NSG-01 from NT-MDT), was used to collect images. The microscope was calibrated using a calibration grating (TGQ1 from NT-MDT) in order to reduce nonlinearity and hysteresis in the measurements. The obtained images were processed with the program Gwyddion, and a statistical analysis as a function of the diameters of 35 different nanoparticles of each type was conducted to compare the results to NTA analysis. Surface properties were analyzed by ζ-potential measurements of the nanoassemblies at pH 7.4 and 25 °C (10 mM NaClO_4_) using dynamic light scattering (DLS) (Nano Zeta Sizer ZS, ZEN3600, Malvern Instruments, Malvern, Worcestershire, UK). Fourier-transform infrared (FTIR) analysis was performed using a FTIR spectrometer (model 6600, Jasco, Japan) equipped with an attenuated total reflection (ATR) diamond crystal window (ATR ProOne). A total of 64 scans were collected in the wavenumber range from 4000 to 400 cm^−1^, at 2 cm^−1^ resolution.

### 2.5. Magnetic Hyperthermia

An AC generator was used to perform the hyperthermic experiments. The experimental setup consisted of induction heating coils made by four turns of water-cooled copper, a power supply, and a chiller to maintain the temperature of the coils. Samples were analyzed at a fixed frequency of 120 kHz and under three magnetic field strengths, 13, 17, and 23 kA/m. Each sample was measured at the center of the coil, with an AC magnetic probe (NanoScience Laboratories Ltd., Staffordshire, UK). All samples were previously pre-thermized at 37.0 ± 0.2 °C. The temperature increase as a function of time was measured with a fiber optic thermometer (Optocon AG, Dresden, Germany), and the specific absorption rate (SAR) and intrinsic loss power (ILP) of the different systems were calculated [32,33], using Equations (1) and (2).

(1) $$SAR=\left(\frac{C\cdot V_{s}}{m}\right)\frac{dT}{dt},$$

(2) $$ILP=\frac{SAR}{fH_{0}^{2}},$$

where *C* is the volumetric specific heat capacity of the sample (*C*_Water_ = 4185 J/LK), *V_s_* is the sample volume (0.2 mL in the reported experiments), and *m* is the mass of solids in the sample (2 mg).

### 2.6. Photothermia

Experiments were run in Eppendorf tubes (0.5 mL) containing 0.2 mL of suspension of the relevant nanoformulation in HEPES buffer. Nanoparticle concentrations were adjusted to [Fe] = 19 mM. During the experiments, each sample was irradiated from the top with a NIR laser (λ = 808 nm) at 0.5, 1, and 2 W/cm^2^ and visualized with a thermography camera (Flir 60 with 320 × 240 pixels, IR resolution, and thermal sensitivity <0.045 °C; FLIR Systems, Inc. Wilsonville, Oregón, USA), in order to measure temperature increases.

### 2.7. Stability and Drug Release

The stability of the TAT–PLGA(DOXO-BMNPs) nanoassembly ([Fe] = 19 mM, 0.2 mL) suspended in HEPES buffer (pH 7.4) or in citrate buffer (pH 5) was evaluated for 7 days (acting as control experiment). In addition, the drug release under acidic conditions in combination with hyperthermia treatment (photothermia (λ = 808 nm, at 2 W/cm^2^) or magnetic hyperthermia (frequency = 120 kHz, H = 23 kA/m)) was evaluated. The nanoformulation was exposed to photothermia treatment for 30 min and to magnetic hyperthermia treatment for 120 min, while DOXO release was indirectly analyzed over the time course experiment from the supernatants as detailed above. At each specific time interval, the nanoassemblies were magnetically separated from the supernatant, and they were resuspended in fresh buffer to continue the remaining time course experiment. Each experiment was performed in triplicate per each condition.

### 2.8. Cell Culture

HepG2 cells from the human hepatoblastoma cell line were supplied by the European Collection of Animal Cell Culture (Salisbury, UK). This cell line was cultured in Minimum Essential Medium (MEM), supplied with 10% heat-inactivated fetal bovine serum (FBS) and 2 mM l-glutamine, and supplemented with 100 U/mL penicillin and 100 μg/mL streptomycin, at 37 °C in a 5% CO_2_ humidified atmosphere. Cell subcultures were performed for testing or maintenance requirements.

### 2.9. Internalization of TAT–PLGA(DOXO-BMNPs)

To assay the amount of internalized BMNPs, HepG2 cells were seeded in 12-well plates (300,000 cells per well) and treated with PLGA(BMNPs) and TAT–PLGA(BMNPs) 300 μg/mL ([Fe] = 3.8 mM) for 48 h. Cells were trypsinized and transferred to 2 mL tubes and centrifuged at 1000 rpm for 5 min. Then, in order to dissolve the cell pellet with internalized nanoparticles, 37% HCl/10% H_2_O_2_ was added and maintained for 20 min at room temperature. Next, 1 mL of 1% potassium thiocyanate in Milli-Q water was added to the tubing, and the absorbance at 490 nm was measured by UV–Vis spectroscopy. To obtain the endogenous iron of the cells, a standard calibration curve was used.

DOXO-bearing experiments are not considered here because of DOXO cytotoxicity and the fact that the protocol used only measures the Fe internalized in viable cells. Therefore, the effects of TAT–PLGA covering and DOXO cytotoxicity cannot be disentangled in DOXO-bearing experiments.

### 2.10. In Vitro Hyperthermia Cytotoxicity Assay

HepG2 cells were seeded on a 96-well plate and grown for 24 h. Then, cells were incubated for 48 h at 37 °C with 30 μg/mL DOXO, TAT–PLGA(DOXO-BMNPs) (containing 300 μg/mL BMNPs and 30 μg/mL DOXO), or just culture medium as a control. After the incubation time, cells were exposed to AMF (frequency = 120 kHz, H = 23 kA/m), and the cell viability was tested as previously reported in [34]. Briefly, formazan crystals generated by MTT (3-(4,5-dimethylthiazol-2-yl)-2,5-diphenyltetrazolium bromide) reaction were solubilized with 100 μL of DMSO, and the absorbance was measured using a microplate reader (HTX Microplate Reader BioTek Instruments, Winooski, VT, USA) at a wavelength of 570 nm.

### 2.11. In Vitro Photothermal Cytotoxicity Assay

The photothermal assay was performed as previously described in [16]. Briefly, HepG2 cells were seeded onto 12 well-plates at 300,000 cells per mL, and, identically to magnetic hyperthermia treatments, cells were incubated for 48 h at 37 °C with 30 μg/mL DOXO, TAT–PLGA(DOXO-BMNPs) (300 μg/mL BMNPs and 30 μg/mL DOXO), or culture medium as a control experiment. After that, cells were tripsinized and resuspended in 200 μL of fresh medium, before being transferred to Eppendorf tubes where they were irradiated for 600 s from the top using an NIR laser (λ = 808 nm) at 2 W/cm^2^. To determine the cell viability, the resazurin assay was done, and the fluorescence was determined at λ_ex_ = 535/λ_em_ = 590 nm in a microplate reader (HTX Microplate Reader BioTek Instruments, Winooski, VT, USA).

### 2.12. Statistical Analysis

Statistical analyses were performed using GraphPad Prism version 8.4.2 for Windows, GraphPad Software (2020, GraphPad Prism, San Diego, CA, USA). For in vitro biological analysis, data represent means ± SEM of three independent experiments performed in triplicate, and statistical analyses were carried out using two-way ANOVA, with a Bonferroni’s post hoc test for grouped analysis. Statistical differences between the treatments were considered significant at *p* ≤ 0.05 (*), *p* ≤ 0.01 (**), and *p* ≤ 0.001 (***).

## 3. Results and Discussion

### 3.1. TAT–PLGA(DOXO-BMNPs) Characterization

**Figure 1:**
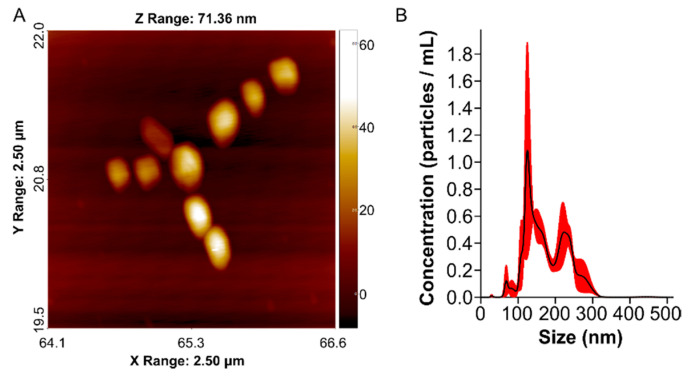
(**A**) AFM analysis and (**B**) NTA size diagram of the TAT–PLGA(DOXO-BMNPs) nanoassembly.

AFM images of TAT–PLGA(DOXO-BMNPs) (Figure 1A) showed nanoassemblies with a spherical shape that exhibited a diameter of 210 ± 50 nm; this size was further confirmed by NTA analysis (175 ± 50 nm). In addition, NTA diagrams showed three different population of nanoparticles (100, 200, and 300 nm), with most of them being 100 and 200 nm, indicating a fairly good colloidal stability and an embedding of a maximum of four DOXO-BMNPs (Figure 1B). The size of this nanoassembly is not significantly different from that determined for PLGA(BMNPs) (204 ± 40 nm (150 ± 50 nm by NTA), Figure S1A).

In the context of surface properties, TAT–PLGA(DOXO-BMNPs) nanoassemblies displayed a ζ-potential at pH 7.4 of −3.8 ± 0.2 mV. At this identical pH, the ζ-potential values of PLGA(BMNPs) and TAT–PLGA(BMNPs) were −32 ± 3 mV and −13 ± 5 mV, respectively [22]. This negative net charge at physiological pH, added to the superparamagnetic character of biomimetic magnetic nanoparticles, avoids the aggregation of the magnetic nanoassemblies in the absence of an external magnetic field due to electrostatic or magnetic dipole–dipole interactions [3,9]. This hypothesis is in agreement with the fairly good colloidal stability shown by DLS and NTA analyses (Figure 1A). On the other hand, the reduction in negative net charge of TAT–PLGA(DOXO-BMNPs) was a consequence of the hiding of the –COOH groups of PLGA polymer during functionalization with the TAT peptide, which also occurred when PLGA(BMNPs) were functionalized with TAT peptide, whereby these –COOH groups were partially neutralized [22]. This result further confirmed the presence of the peptide on the nanoformulation surface.

Concerning the nanoassembly composition, FTIR data (Figure S2) showed adsorption bands that were different in BMNPs and TAT–PLGA(DOXO-BMNPs) nanoassemblies, further confirming the coupling. The peak at 542 cm^−1^, characteristic of the Fe–O bond in magnetite nanoparticles, was found in both spectra, but not in the DOXO spectrum. However, the FTIR spectrum of TAT–PLGA(DOXO-BMNPs) showed additional peaks in the ranges 900–1800 and 2700–3500 cm^−1^. In this range, the TAT–PLGA(DOXO-BMNPs) spectrum showed the characteristic C=O peak of the carboxyl group at 1749 cm^−1^. In addition, in these two ranges, there were peaks corresponding to the DOXO molecule. Lastly, regarding the TAT peptide presence, there were peaks corresponding to amide I, II, and III bond vibration.

### 3.2. TAT–PLGA(DOXO-BMNPs) Nanoassemblies as Hyperthermic Agents

#### 3.2.1. Magnetic Hyperthermia

**Figure 2:**
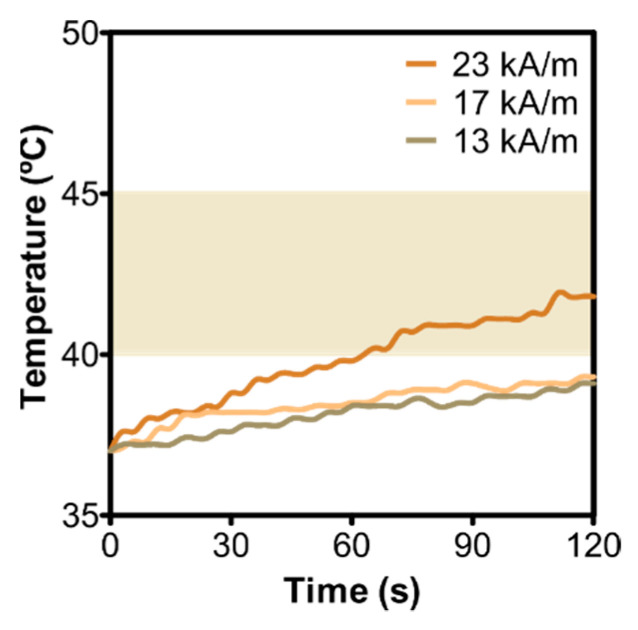
Time evolution of the temperature increase caused by TAT–PLGA(DOXO-BMNPs) nanoassemblies following exposure to AMF of different magnetic field strengths at a fixed frequency of 120 kHz.

Following exposure to an alternating magnetic field (AMF) of 23 kA/m and a frequency of 120 kHz, TAT–PLGA(DOXO-BMNPs) nanoassemblies raised the temperature to the effective hyperthermia temperature (42–46 °C) within ~70 s (Figure 2). SAR and ILP values for this nanoassembly were 24 ± 2 W/g and 0.37 ± 0.03 nH m^2^/kg, respectively (Table S1). These values were lower than those displayed by the PLGA(BMNPs) nanoassembly (68 ± 5 W/g and 1.06 ± 0.8 nH m^2^/kg, respectively) (Table S2). The same output occurred when the magnetic field was varied within the range 13 to 23 kA/m at a fixed frequency of 120 kHz. Within 120 s of exposure, only TAT–PLGA(DOXO-BMNPs) nanoassemblies exposed to AMF of 23 kA/m were able to reach hyperthermia temperatures. On the contrary, when PLGA(BMNPs) were used instead, an AMF higher than 17 kA/m was enough to raise the temperature to therapeutic values (Figure S3). This loss of magnetic response is consistent with the shelter resulting from the encapsulation of the magnetic BMNPs by a nonmagnetic coat (DOXO and TAT–PLGA, in this case), as extensively reported both for inorganic magnetic nanoparticles [6] and for biomimetic magnetic nanoparticles [22]. Moreover, the PLGA covering may pose restrictions for the rotation of the BMNPs to align with the external AMF, thus reducing Brownian relaxation, which is one of the main causes of heating [9]. Nevertheless, in spite of the partial loss of a magnetic hyperthermic response, our results show that TAT–PLGA(DOXO-BMNPs) nanoassemblies were still able to induce heating to hyperthermia temperatures when subjected to an AMF by using magnetic field strengths and frequencies clinically acceptable [35,36].

#### 3.2.2. Photothermia

**Figure 3:**
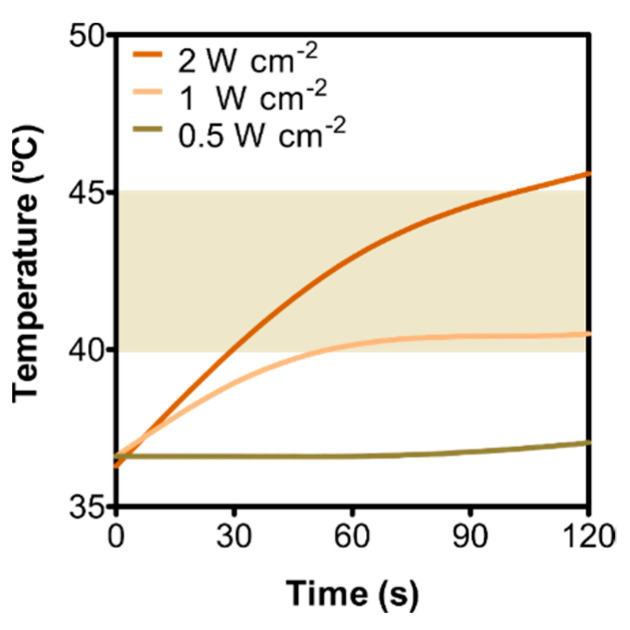
Time evolution of the temperature of TAT–PLGA(DOXO-BMNPs) suspension at different laser power densities.

Following irradiation of TAT–PLGA(DOXO-BMNPs) nanoassemblies with an NIR laser (λ = 808 nm), the temperature of the nanoassembly suspensions increased proportionally to the applied laser power (Figure 3, Table S3). When a laser power density of 2 W/cm^2^ was applied, the nanoassemblies were able to reach the therapeutic temperature in 30 s, whereas, when this laser power density decreased to 1 W/cm^2^, it took ~50 s to reach such therapeutic temperatures. The temperature rise caused by the TAT–PLGA(DOXO-BMNPs) nanoassembly was significantly lower than that caused by PLGA(BMNPs) (Figure S4, Table S3), probably related to the smallest (nonfunctionalized) BMNP area exposed to irradiation, as well as to heat losses within the different coatings (DOXO, PLGA, and TAT). However, it is important to note that, in spite of all these irradiation barriers, TAT–PLGA(DOXO-BMNPs) nanoassemblies were able to reach the hyperthermia temperature in less than a minute in irradiation conditions that avoid collateral tissue damage in vivo [13].

### 3.3. TAT–PLGA(DOXO-BMNPs) Nanoassemblies as Nanocarriers

**Figure 4:**
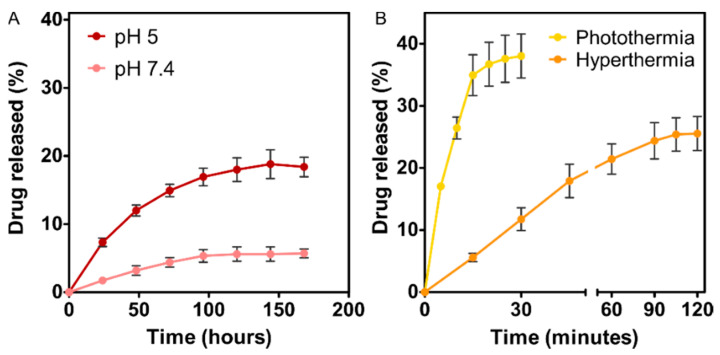
TAT–PLGA(DOXO-BMNPs) as drug nanocarriers. DOXO release at physiological and acidic pH values in (**A**) absence of magnetic hyperthermia and/or photothermia. (**B**) DOXO release at acidic pH in combination with photothermia (2 W/cm^2^) or magnetic hyperthermia (frequency = 120 kHz, H = 23 kA/m) treatment.

The ability of the nanoassembly to behave as a drug nanocarrier was evaluated at pH 7.4 (physiological pH) and pH 5 (mimicking the environment in the endosomal/lysosomal compartment [37]) (Figure 4A).

DOXO release from the nanoassembly at physiological pH was very low, with release efficiency (D_R_) values that did not exceed 6% of the adsorbed drug after 7 days. This result indicates the fairly good stability of the nanoformulation at physiological pH values, as previously proven by other studies [3,11]. When the environmental pH value turned acidic, ~20% of the DOXO was released from the nanoassembly within the first 24–48 h, which is consistent with previous findings [11,12]. Since the binding of DOXO to BMNPs is mediated by electrostatic interactions, when the environment pH approaches the isoelectric point of the BMNPs (pH 4.3), the BMNPs become uncharged, thus releasing the drug. It is interesting to note that the D_R_ coefficient in the present experiments was lower than that obtained using DOXO-BMNPs (35% [3]). This lower D_R_ value is consistent with the partial trapping of DOXO by PLGA. Since DOXO is positively charged at acidic pH values [38], it may get entrapped by the –COOH groups of PLGA that are not bound to TAT peptide, thus decreasing release to the outer bulk medium.

However, when this experiment of DOXO release at acidic pH values was combined with photothermia or magnetic hyperthermia, D_R_ values increased to 25% and to 35% within the first 90 and 15 min, respectively (Figure 4B). The enhanced DOXO release in a short period of time was the consequence of the synergy resulting from the combination of the reduced electrostatic affinity between DOXO and the almost neutral BMNPs, and the increment of the BMNP thermal energy induced by the external stimuli. Moreover, once DOXO is released from the BMNP, the temperature rise may, on one hand, open the structure of PLGA [31], causing the DOXO release to the bulk solution, and, as observed for the DOXO-BMNP nanoassembly [3], it may reduce the stability of the electrostatic binding between DOXO and the carboxylic groups of PLGA, all of which favor DOXO release into the bulk medium. This is important, since the fact that the nanoassembly responds to active/external (photothermia or magnetic hyperthermia) and passive/endogenous (acidic pH) stimuli would allow a spatiotemporally controlled drug delivery strategy.

### 3.4. Cell Uptake

**Figure 5:**
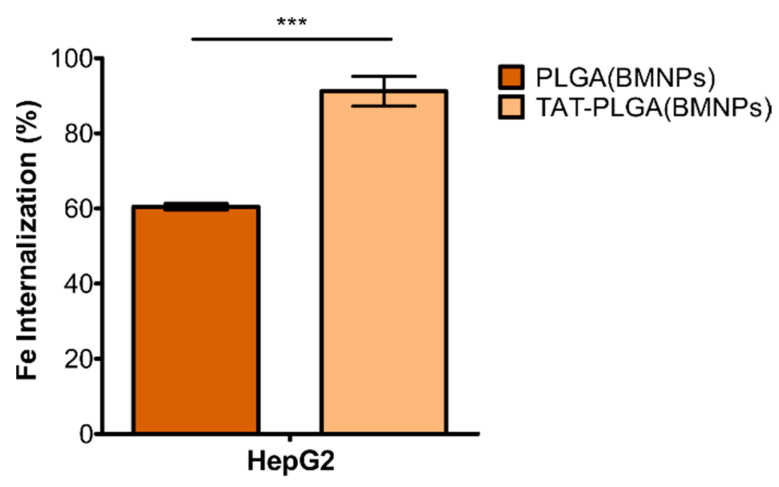
Internal iron content in the viable cells upon treatment with PLGA(BMNPs) and TAT–PLGA(BMNPs). Data represent the means ± SEM of three independent experiments performed in triplicate; *p* ≤ 0.001 (***).

Previous studies have shown that both BMNPs and PLGA(BMNPs) are internalized via endocytosis when they are incubated with cells [16,22,34]. The cellular uptake was determined by estimating the Fe internalized into the cells after their incubation with the different nanoformulations (PLGA(BMNPs) and TAT–PLGA(BMNPs)). After 48 h of incubation, an internalization of 60% PLGA(BMNPs) and 91% TAT–PLGA(BMNPs) was observed, with these differences being statistically significant (Figure 5). Therefore, the presence of the TAT moiety facilitated internalization of the nanoformulation into the cells [39,40,41].

### 3.5. TAT–PLGA(DOXO-BMNPs) Cytotoxicity Combined with Magnetic Hyperthermia or with Photothermia

**Figure 6:**
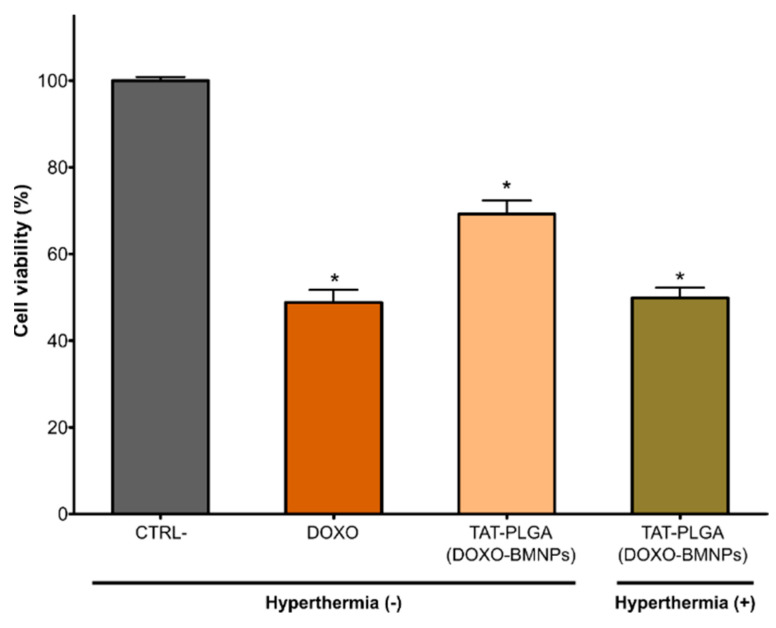
Cytotoxicity in HepG2 cell line following treatment with culture medium (control), DOXO (30 μg/mL DOXO), and TAT–PLGA(DOXO-BMNPs) (containing 300 μg/mL BMNPs and 30 μg/mL DOXO) in the absence or in combination with magnetic hyperthermia. Data represent the means ± SEM of three independent experiments performed in triplicate; *p* ≤ 0.05 (*).

HepG2 viability was significantly reduced, by up to 70%, after treatment with TAT–PLGA(DOXO-BMNPs) for 48 h (Figure 6). This decrease in cell viability is not as high as that produced by soluble DOXO (~50%). However, when this treatment was combined with magnetic hyperthermia by exposing the cell to an alternating magnetic field (frequency = 120 kHz, H = 23 kA/m), cell viability was fairly similar to that caused by soluble DOXO (Figure 6). This further reduction in cell was probably caused by the increase in DOXO released triggered by magnetic hyperthermia (Figure 4B) combined with the increase in temperature mediated by the nanoassembly (Figure 2). In addition, when the nanoassembly is internalized by the cell, the PLGA envelope starts decomposing, as it is used by cell metabolism, thus, on one hand, releasing the DOXO-BMNPs and, on the other, creating an acidic environment in the cell caused by the release of the acids that integrate the PLGA [42,43]. This acidification would further trigger the DOXO release from the nanoassembly, potentiating the cytotoxic effect.

**Figure 7:**
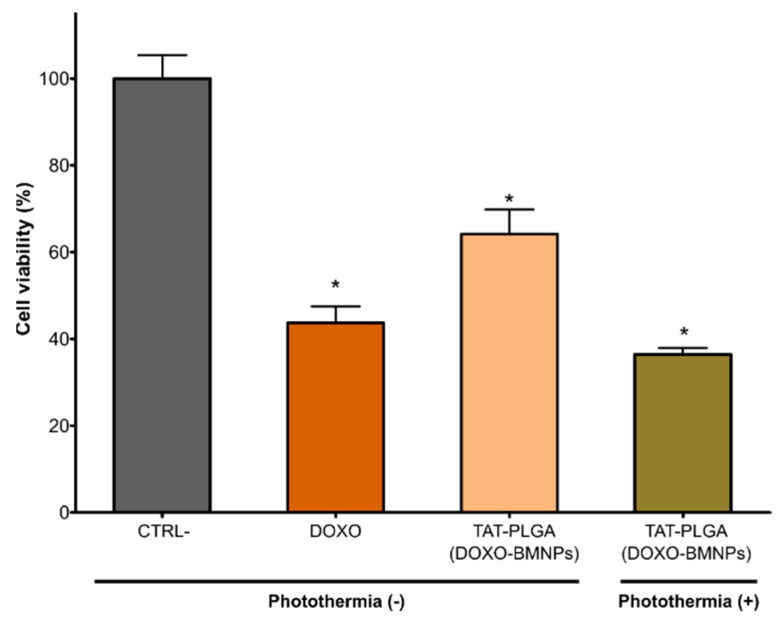
Cytotoxicity in HepG2 cell line cell line following treatment with culture medium (control), DOXO (30 μg/mL), and TAT–PLGA(DOXO-BMNPs) (containing 300 μg/mL BMNPs and 30 μg/mL DOXO) in the absence or presence of photothermia. Data represent the means ± SEM of three independent experiments performed in triplicate; *p* ≤ 0.05 (*).

Identically, after the exposure of TAT–PLGA(DOXO-BMNPs) to laser powers of 2 W/cm^2^ for 600 s (Figure 7), a significant reduction in HepG2 cell viability (up to 65% of cell viability), even higher than that caused by soluble DOXO, was observed (Figure 6). As earlier, both the temperature increases (Figure 3) and the highest DOXO release observed following combination with photothermia (Figure 4B) may have accounted for the higher cytotoxicity of the TAT–PLGA(DOXO-BMNPs) nanoassembly.

These results are promising, since they demonstrate that the TAT–PLGA(DOXO-BMNPs) nanoassembly, when exposed to an AMF or, even better, to photothermia, could be able to reach a comparable (or stronger) antiproliferative efficiency to that caused by soluble DOXO, but through a directed local versus systemic therapy.

## 4. Conclusions

The results from the present study demonstrate that DOXO-BMNP nanoassemblies may be embedded in PLGA and further functionalized with the cell-penetrating TAT peptide, while maintaining their potential as magnetic hyperthermic and photothermic agents. The TAT–PLGA(DOXO-BMNPs) nanoassembly showed a fairly good colloidal stability, permitted by the negatively charged surface of the nanoassembly and the superparamagnetic characteristic of the BMNPs, thus preventing electrostatic and/or magnetic dipole/dipole interactions. The size of the nanoassemblies (most of them being 100 and 200 nm) showed that they embedded a maximum of four DOXO-BMNPs nanoparticles. This TAT–PLGA(DOXO-BMNPs) nanoassembly was able to mediate both directed chemotherapy and hyperthermia treatment (either magnetic hyperthermia or photothermia) using the same nanoplatform. While the TAT–PLGA(DOXO-BMNPs) nanoassembly is able to reduce cell viability, its cytotoxic effect is similar (or stronger) when directed chemotherapy is combined with magnetic hyperthermia or with photothermia. This synergy is caused by the enhanced DOXO release following treatment combination and the locally induced temperature increase. Therefore, the results of this work represent a step forward in the use of combined therapies to increase the antitumor efficiency of treatments, as well as a transition from systemic to local treatments with the goal of reducing drug doses and undesirable secondary effects.

## Data Availability

Not applicable.

## Supplementary Materials

The following are available online at https://www.mdpi.com/article/10.3390/pharmaceutics13081168/s1: Figure S1. (A) AFM analysis and (B) NTA size diagram of the PLGA(BMNPs) nanoassembly; Figure S2. FT-IR analysis of BMNPs, TAT-PLGA(DOXO-BMNPs) and DOXO; Figure S3. Time evolution of the temperature increase caused by PLGA(BMNPs) nanoassemblies following exposure to AMF of different magnetic field strengths at a fixed frequency of 120 kHz; Figure S4. Time evolution of the temperature of PLGA(BMNPs) suspension at different laser power densities; Table S1. Summary of SAR and ILP calculations concerning TAT–PLGA(DOXO-BMNPs) at different intensities and at a fixed frequency of 120 kHz, after 30 s of exposure. Sample volume, 0.2 mL; particle concentration, 10 mg/mL; Table S2. Summary of SAR and ILP calculations concerning PLGA(BMNPs) at different intensities and at a fixed frequency of 120 kHz, after 30 s of exposure. Sample volume, 0.2 mL; particle concentration, 10 mg/mL; Table S3. Summary of SAR calculations at different laser power densities (0.5, 1, and 2 W/cm^2^), after 120 s of exposure. Sample volume, 0.2 mL; particle concentration, 19 mM.
