# Supplementary material for: Enhanced Cytotoxic Effect of TAT–PLGA-Embedded DOXO Carried by Biomimetic Magnetic Nanoparticles upon Combination with Magnetic Hyperthermia and Photothermia
Source: Pharmaceutics. 2021 Jul 28;13(8):1168. doi: 10.3390/pharmaceutics13081168 (PMC8398382; doi:10.3390/pharmaceutics13081168)
Supplement: Supplementary file 1 [file pharmaceutics-13-01168-s001.zip › pharmaceutics-1235282-supplementary.pdf]

pharmaceutics

Supplementary Materials: Enhanced Cytotoxic Effect of TAT–PLGA-Embedded DOXO Carried by Biomimetic Magnetic Nanoparticles upon Combination with Magnetic Hyperthermia and Photothermia

Ylenia Jabalera, Alberto Sola-Leyva, Salvatore Calogero Gaglio, María P. Carrasco Jimenez, Guillermo R. Iglesias, Massimiliano Perduca and Concepción Jimenez-Lopez

Table S1. Summary of specific absorption rate (SAR) and intrinsic loss power (ILP) calculations concerning TAT-PLGA(DOXO-BMNPs) at different intensities and at fixed frequency of 120 kHz, after 30 seconds of exposure. Sample volume 0.2 ml; particles concentration of 10 mg/ml.

Nanoformulation	Field [kA/m]	SAR [W/g]	Slope dT/dt [°C/s]	ILP nH m²/kg
TAT-PLGA(DOXO-BMNPs)	13.2 ± 0.2	7.4 ± 0.9	0.01	0.36 ± 0.05
	17.8 ± 0.2	17.0 ± 2.0	0.04	0.48 ± 0.06
	23.3 ± 0.2	24.0 ± 2.0	0.056	0.37 ± 0.03

Table S2. Summary of SAR and ILP calculations concerning PLGA(BMNPs) at different intensities and at fixed frequency of 120 kHz, after 30 seconds of exposure. Sample volume 0.2 ml; particles concentration of 10 mg/ml.

Nanoformulation	Field [kA/m]	SAR [W/g]	Slope dT/dt [°C/s]	ILP nH m²/kg
PLGA(BMNPs)	13.4 ± 0.2	10 ± 1	0.02	0.46 ± 0.05
	17.7 ± 0.2	24 ± 2	0.06	0.72 ± 0.04
	23.2 ± 0.2	68 ± 5	0.16	1.06 ± 0.80

Table S3. Summary of SAR calculations concerning PLGA(BMNPs) and TAT-PLGA(DOXO-BMNPs) at different laser power densities (0.5, 1 and 2 W/cm²), after 120 seconds of exposure. Sample volume 0.2 ml; particles concentration of 19 mM.

Nanoformulation	Laser Power Density [W/cm²]	Slope dT/dt [°C / s]
PLGA(BMNPs)	0.5 ± 0.1	0.024 ± 0.003
	1.0 ± 0.1	0.045 ± 0.002
	2.0 ± 0.1	0.120 ± 0.020
TAT-PLGA(DOXO-BMNPs)	0.5 ± 0.1	0.008 ± 0.001
	1.0 ± 0.1	0.029 ± 0.004
	2.0 ± 0.1	0.080 ± 0.010

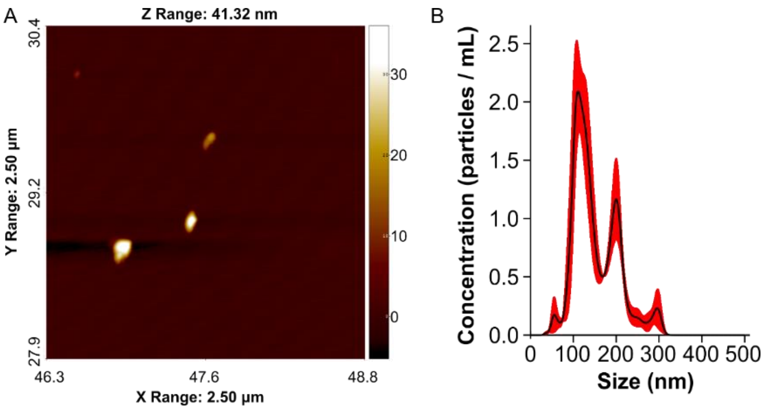

Figure S1. (**A**) AFM analysis and (**B**) NTA size diagram of the PLGA(BMNPs) nanoassembly.

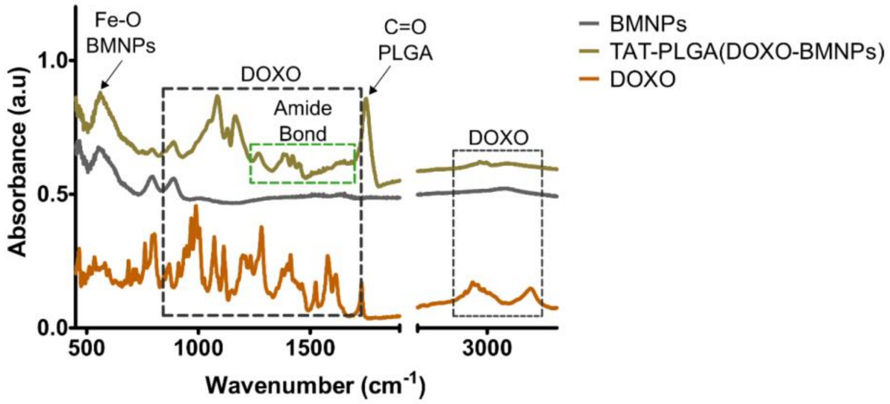

Figure S2. FT-IR analysis of BMNPs, TAT-PLGA(DOXO-BMNPs) and DOXO.

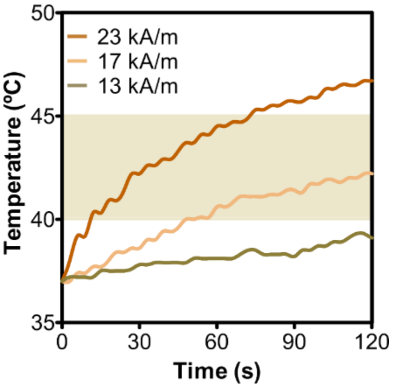

Figure S3. Time evolution of the temperature increase caused by PLGA(BMNPs) nanoassemblies following upon exposure to AMF of different magnetic field strengths at a fixed frequency of 120 kHz.

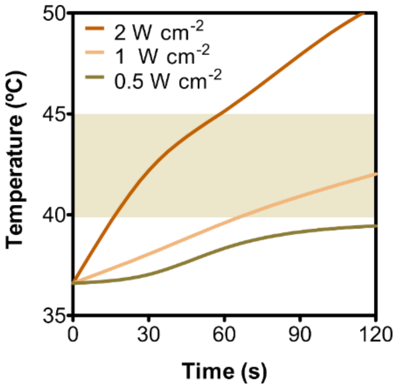

Figure S4. Time evolution of the temperature of PLGA(BMNPs) suspension at different laser power densities.
